# Knockdown of circROBO2 attenuates acute myocardial infarction through regulating the miR-1184/TRADD axis

**DOI:** 10.1186/s10020-021-00275-6

**Published:** 2021-03-03

**Authors:** Tian-ping Chen, Nai-ju Zhang, Hong-ju Wang, Si-gan Hu, Xu Geng

**Affiliations:** Department of Cardiology, the First Affiliated Hospital of Bengbu Medical College, No. 287, Changhuai Road, Longzi Lake District, Bengbu city, 233003 Anhui province China

**Keywords:** circROBO2, miR-1184, TRADD, Acute myocardial infarction, Apoptosis

## Abstract

**Background:**

Studies have found that circular RNAs (circRNAs) play key roles in cardiovascular diseases. However, the function of circROBO2 in acute myocardial infarction (AMI) is unclear. This study aimed to investigate the pathogenesis of circROBO2 in AMI.

**Methods:**

qRT-PCR and Western blot were used to determine the expression levels of circROBO2, miR-1184, and TRADD in AMI and sham-operated mouse models at mRNA and protein level, respectively. The relationship among miR-1184, circROBO2 and TRADD was evaluated by RNA immunoprecipitation (RIP) analysis and luciferase reporter gene analysis. The roles of circROBO2, miR-1184, and TRADD in myocardial cell apoptosis were evaluated using flow cytometry. Ultrasound echocardiography, serum creatine kinase MB (CK-MB) and lactate dehydrogenase (LDH), myocardial infarction area, and myocardial cell apoptosis were measured to examine the effects of circROBO2 on myocardial injury.

**Results:**

The expression levels of miR-1184 were significantly reduced, and the expression levels of circROBO2 and TRADD were significantly increased in MI group. CircROBO2 acted as a sponge for miR-1184 by upregulating the expression of TRADD. In addition, overexpression of miR-1184 enhanced the protective effect of knockdown of circROBO2 by partially inhibiting the expression of TRADD in vivo and in vitro.

**Conclusion:**

Knockdown of circROBO2 reduced the apoptosis of cardiomyocytes by increasing the expression levels of miR-1184, which in turn decreased the expression levels of TRADD in the myocardium post-MI.

## Introduction

Acute myocardial infarction (AMI) is a major cause of ischemic cardiomyopathy (Worner et al. 2013). Although its incidence has declined in high-income regions, the global aging and population growth have led to an increasing number of people suffering from atherosclerotic vascular disease (Orti et al. 2007; Anderson and Morrow 2017). The occurrence of myocardial infarction is due to long-term ischemia of myocardial cells, leading to myocardial cell death, and the risk of death is the highest within a few hours of AMI (Heusch and Gersh 2017; Thiele et al. 2017). Therefore, diagnosis at early stage of AMI is crucial for effective treatment of AMI patients (Lippi et al. 2013). In addition, although great progress has been made in the treatment of AMI, the diagnosis of myocardial infarction is still challenging for patients with unstable angina pectoris (UA) symptoms, and there is still residual risk of mortality and morbidity (Kelm et al. 2018). Troponin I is an ideal marker for myocardial injury. However, the clearance time is too long and it is difficult to identify recurrent myocardial infarction (Labugger et al. 2000). Serum CK-MB in chest pain starts to rise at 4–9 h after the occurrence, which is also too late for AMI. Early myocardial infarction is difficult to be identified, so it is important to find new myocardial markers (Chon et al. 2013).

Circular RNAs (circRNAs) are widely present in mammalian cells and involved in regulation of gene expression (Gong et al. 2018; Li et al. 2019a). With the development of RNA sequencing technologies and biophysical technology, extensive studies have investigated the roles of circRNAs in gene regulation and disease occurrence (Yang et al. 2019; Wang et al. 2019). Recent studies have shown that some circRNAs are involved in the development of various cardiovascular diseases, including myocardial infarction, cardiac metabolic disease and heart failure (Jiang et al. 2016; Fan et al. 2017). And the potential roles that circRNAs play in cardiovascular disease have been identified (Corsten et al. 2010). Therefore, the regulation of circRNAs might serve as a biomarker for the treatment of this disease (Wang et al. 2017). CircROBO2 is a newly identified circRNA and its role in AMI remains unclear.

In recent years, the regulatory relationship between circRNAs and miRNAs has attracted extensive attention (Lin et al. 2018). MiRNAs are one of the most characterized targets of circRNAs (Xiao (2019)). It is well known that miRNAs are involved in the physiological and pathophysiological processes of cellular signals (Lerner et al. 2014). Regulation of miRNAs in AMI samples may help to reduce apoptosis, protect tissues from damage, promote new blood vessel formation, control the degree of wall fibrosis, and thereby improving long-term prognosis (Yan et al. 2017). Many miRNAs can directly regulate downstream target proteins to aggravate the pathological process of myocardial infarction, and intervention of miRNA signaling pathways can provide new strategies for the clinical treatment of AMI (Sun et al. (2018)). MiR-1184 participates in multiple malignant transformation processes in cells, and abnormal expression of miR-1184 has been reported in many diseases (Knyazev et al. 2016). However, its function in AMI and the regulatory mechanisms are not clear. TNFR1-associated death domain protein (TRADD), encoded by the TRADD gene, is an adaptor protein in the human genome (Jackson-Bernitsas and Ichikawa 2007). TRADD is the first identified protein related to tumor-necrosis factor receptor-1 (TNFR1), which is commonly found in most body tissues. Downregulation of TRADD largely protects tumor cells from undergoing apoptosis (Zheng et al. 2006). Studies have found that TRADD plays a key role in cardiovascular disease (Hostiuc et al. 2013). Therefore, we speculated that circROBO2 may regulate the progress of AMI through the miR-1184/TRADD axis. The main purpose of this study was to explore the mechanism of circROBO2 in regulating AMI and to provide theoretical basis for finding new drug targets.

## Materials and methods

### Mouse MI model

A total of 100 male C57B/L6 mice aged 8–10 weeks and weighed 18–25 g were purchased from the First Affiliated Hospital of Bengbu Medical College Animal Research Institute. The mice were anesthetized by intraperitoneal injection of avertin solution before surgery, fixed in supine position on the mouse plate. The MI group included 80 mice and the sham group included 20 mice. The ventilator was ligated to the anterior descending branch of the left coronary artery 3–4 mm as previously described (Akodad et al. 2017). There were five mice died in the MI group 3 days after the surgery, and no mice died in the sham group. The myocardial tissues of ten mice from the MI group and sham group were harvested. In the following 4 weeks, ten mice in the MI group died, and the rest were bred for subsequent experiments.

### Recombinant adeno-associated virus 9 (rAAV9)-mediated gene delivery in the heart

The rest mice which had the operation were randomly selected and divided into five groups (n = 10) including the sham group, MI group, rAAV9-si-circROBO2 group (si-circROBO2), rAAV9-miR-1184 group (miR-1184), and combined group (expressing si-circROBO2 and miR-1184). Then, 20 μl containing 5 × 10^12^ GC of rAAV9 vectors were injected in five randomly selected sites within left ventricles. Mice in the sham and MI groups were injected with rAAV9 vectors. The expression levels of circROBO2 and miR-1184 in myocardium were identified 1 week after injection.

### HE &TTC histology staining

The heart was excised and sliced horizontally into 6–7 slices from base to apex. Samples were incubated in 1% 2,3,5-triphenyl-tetrazolium chloride (TTC; Sigma-Aldrich, USA) diluted in PBS (pH 7.4) at 37 °C for 30 min. Stained myocardial samples were photographed from both sides, and in each slide, the infarct area was compared to the total left ventricular area. Six of random pictures were taken for each slide at × 4 magnification for measurement of infarct size.

Each heart was fixed in 4% paraformaldehyde for at least 30 min and cut into 5 μm sections. Slides were then stained as following: 70% ethyl alcohol (EtOH) for 10 s, water for 5 s, hematoxylin for 10 s, 70% EtOH for 30 s, eosin for 20 s, and xylenes for 2 min. Finally, after being washed for three times, the histological structure of myocardial tissues was observed with a microscope (× 100).

### Immunohistochemistry and immunofluorescence

Paraffin-embedded myocardial tissue sections were dewaxed in xylene and hydrated in ethanol. Tissue sections were then incubated in 30% H2O2 for 30 min. After 10 min of antigen recovery in 10 mm citrate buffer, tissue sections were incubated with anti-TRADD primary antibody (1:100; Thermo Fisher), Caspase 3 (1:1000; Thermo Fisher) and TUNEL (1:500; Thermo Fisher) for overnight. The corresponding mouse horseradish peroxidase (HRP)-conjugated secondary antibody (1:500; Thermo Fisher) was then added. The image was observed under an optical microscope.

Serial sections (5 μm) of the heart from each group were selected for immunofluorescence staining. Sections were blocked with 5% goat serum before incubating with rabbit anti-nkx2.5 (nkx2.5, Abeam, 0.5 μg/ml) primary antibodies, followed with incubation with a recombinant terminal deoxynucleotidyl transferase (rTdT) solution for 1 h. Goat anti-rabbit Alexa fluor595 or goat anti-rabbit Alexa fluor532 were used as secondary antibodies for probing TUNEL and goat anti-rat Alexa fluor633 secondary antibody for nkx2.5.

### RNA ISH

In situ detection of circROBO2 transcription was performed using the RNAscope kit (Advanced Cell Diagnostics, Hayward, CA, USA). A horseradish peroxidase-based signal amplification system was used for hybridization to the target probes, followed by color development with 3,3′-diaminobenzidine. Positive staining was determined by brown punctate dots in the nucleus and/or cytoplasm.

### Dual luciferase reporter assay

Circ Interactome (https://circinteractome.nia.nih.gov/index.html) and TargetScan v7.2 were used to predict the interaction among circROBO2, miR-1184 and TRADD. The wild type or mutant circROBO2 in combination with miR-1184 were inserted into the pMIR Basic vector (OBiO Biology, Shanghai), and the vector was designated as pMIR-REPOR-circROBO2-wt or pMIR-REPOR-circROBO2-mt. After 24 h of culturing, miR-1184 mimic or mock control, or miR-1184 inhibitor or inhibitor NC (GenePharma, Shanghai, China) were used to transfect cells and co-transfected with empty pMIR. The luciferase activity was measured 48 h after transfection using a dual luciferase assay system (Promega).

### RNA immunoprecipitation

RNA immunoprecipitation was performed using EZMagna RIP kit (Millipore). Cells were lysed using complete RIP lysis buffer. Magnetic beads conjugated with anti-AGO2 or anti-IgG antibodies were incubated with cell extracts. The cells extracts were incubated for 6 h and RNAs were isolated for RT-qPCR.

### Cell culture and isolation

Cardiomyocytes were isolated from newborn mice. The whole skin of the suckling mice was disinfected, the sternum was cut open, the heart was squeezed out, and tissues around the apex were cut into the pre-chilled D-Hanks solution and rinsed for three times. Myocardium was cut into 0.5–1 mm^3^ and 0.08% trypsin solution was added, followed by digestion on a magnetic stirrer for 4 min. The supernatant was then discarded, and 0.1% type I collagenase solution was added for digestion. The supernatant was transferred into a sterile centrifuge tube, and DMEM culture solution was added to stop the digestion. The supernatant was then discarded and DMEM culture solution was added to the pellet. After repeated pipetting, the cell suspension was transferred to sterile culture. After incubation in the dish for 90 min, the petri dish was taken out. The non-adherent cell suspension was inoculated at a density of 2 × 10^5^/mL into a 6-well plate, and BrdU was added at a final concentration of 0.1 mmol/L to inhibit fibroblasts. Then it was placed in a cell incubator for 48–60 h. The solution was firstly changed without adding Brd U, and the solution was changed every other day.

### Cell transfection

The pcDNA-circROBO2(circROBO2), circROBO2 siRNA(si-circROBO2), miR-1184 mimic, miR-1184 agomir, TRADD siRNA (si-TRADD), miRNA negative control (miR-NC), or negative control were purchased from GenePharma Co., Ltd. (Shanghai, China). Mouse cardiomyocytes were placed in 24-well plates and cells were transiently transfected with RNAiMax and Lipofectamine 3000 with Plus Reagent (Thermo Fisher Scientific). Transfection efficiency was determined by qRT-PCR.

### Induction of myocardial hypoxia and cell therapy

Cardiomyocytes used in the experiment were all cultured in 50 ml culture flasks. After 24 h of culturing, 2.5 ml DMEM medium and 47.5 ml PBS medium containing 20% calf serum were added, and the hypoxia gas containing 5% CO2 and 95% N2 was introduced for 2 min (gas flow rate 0.5 L/min). The pH of the culture solution was stabilized at about 7.4, and oxygen in the remaining space was eliminated. The culture bottle cover was caped and sealed with wax and placed in a CO_2_ incubator at 37 °C.

### CCK-8 assay

Cells (2 × 10^4^ cells/ml) were seeded into 96-well plates and placed in a cell incubator. After 2 days of culturing, 10 μl of CCK8 solution was added. The absorbance values at 450 nm were measured to assess cell viability.

### Annexin V-FITC/PI assay

Annexin V-FITC apoptosis kit was used to analyze cardiomyocyte apoptosis. Transfected cells were harvested, and the Annexin V-FITC-PI detection kit was used following the manufacturer's instructions (Haigene, Haerbin, China). Cell apoptosis was analyzed by FACScan flowcytometer (BD Biosciences, San Jose, CA, USA).

### RNA extraction and quantitative real-time PCR

Total RNAs in cells were extracted using TRIzol reagent (Invitrogen, Carlsbad, CA). After reverse transcription reaction, qRT-PCR was performed using ViiA™ 7 real-time PCR system (Life Technologies, Grand Island, NY). GAPDH and U6 were used as the internal references. The expression levels of circRNA/mRNA and miRNA were calculated using the 2^−ΔΔCT^ method. All experiments were repeated for three times. qRT-PCRs were performed as described in literature (Mehta et al. 2010). The primer sequences were listed in Table 1.

**Table 1:** Primers sequences used in this study

Name	Sequence (5′–3′)
circROBO2	Forward: TCTGGTATTGCCTGGAACGCCAA
	Reverse: CAGTGGTAATCAAGTGGAGAATC
ROBO2	Forward: ACCTTTGGGTGGGGAAGTGA
	reverse: GCAACACCTCGGTATACCGA
TRADD	Forward: GAGCCACTTAGAATCGAGGA
	Reverse: CTGAGGCTATAGATTCGTGCC
miR-1184	Forward: GAGCTAGCGAATGGCACCCT
	Reverse: GCAGGAACGAAGTCGACTTA
si-TRADD	Sense: ATCTTGAGCCATTCACCGGAA
si-circROBO2	Sense: AGGCTGAGCTAATACAGTA
U6	Forward: AGAGAAGATTAGCATGGCCCCTG
	Reverse: AGTGCAGGGTCCGAGGTATT
GAPDH	Forward: CCAAGGTCATCCATGACAAC
	Reverse: GCTTCACCACCTTCTTGATG

### Western blot

The transfected cells were collected and total proteins were extracted. Protein concentration was determined using the BCA Protein Assay Kit. Protein samples were separated on SDS-PAGE and then transferred to a polyvinylidene fluoride (PDVF) membrane. The membrane was incubated with anti-TRADD antibodies (1:1000, Proteintech, Chicago, IL, USA) and anti-GAPDH antibodies (1:1000, Abcam, Cambridge, UK) at 4 °C for overnight. Then the membrane was incubated with 1:5000 labeled anti-rabbit secondary antibody for 1 h. Western blot analysis was performed with reference to the literature (Sanchez-Campillo et al. 2015).

### Echocardiographic evaluation

Cardiac function was assessed by chest echocardiography 1 d before mouse was euthanized. M-mode echocardiography was used to measure echocardiographic parameters including left ventricular ejection fraction (LVEF), left ventricular shortening fraction (LVFS), left ventricular end-systole Diameter (LVESd) and left ventricular end-diastolic diameter (LVEDd).

### Determination of serum CK-MB and LDH

Serum creatine kinase MB (CK-MB) and lactate dehydrogenase were measured on an automatic biochemical analyzer using a creatine kinase activity assay kit and an LDH colorimetric assay kit (Jiancheng Biotechnology Research Institute, Nanjing, China).

### Statistical analysis

Differences among multiple groups were compared by Kruskal–Wallis test. All data were analyzed using the SAS software (version 9.0; SAS Institute, Cary, NC, USA). A *P*-value < 0.05 was considered as statistically significant.

## Results

### CircROBO2 was up-regulated in MI mouse model and hypoxia-treated cardiomyocytes

**Fig. 1:**
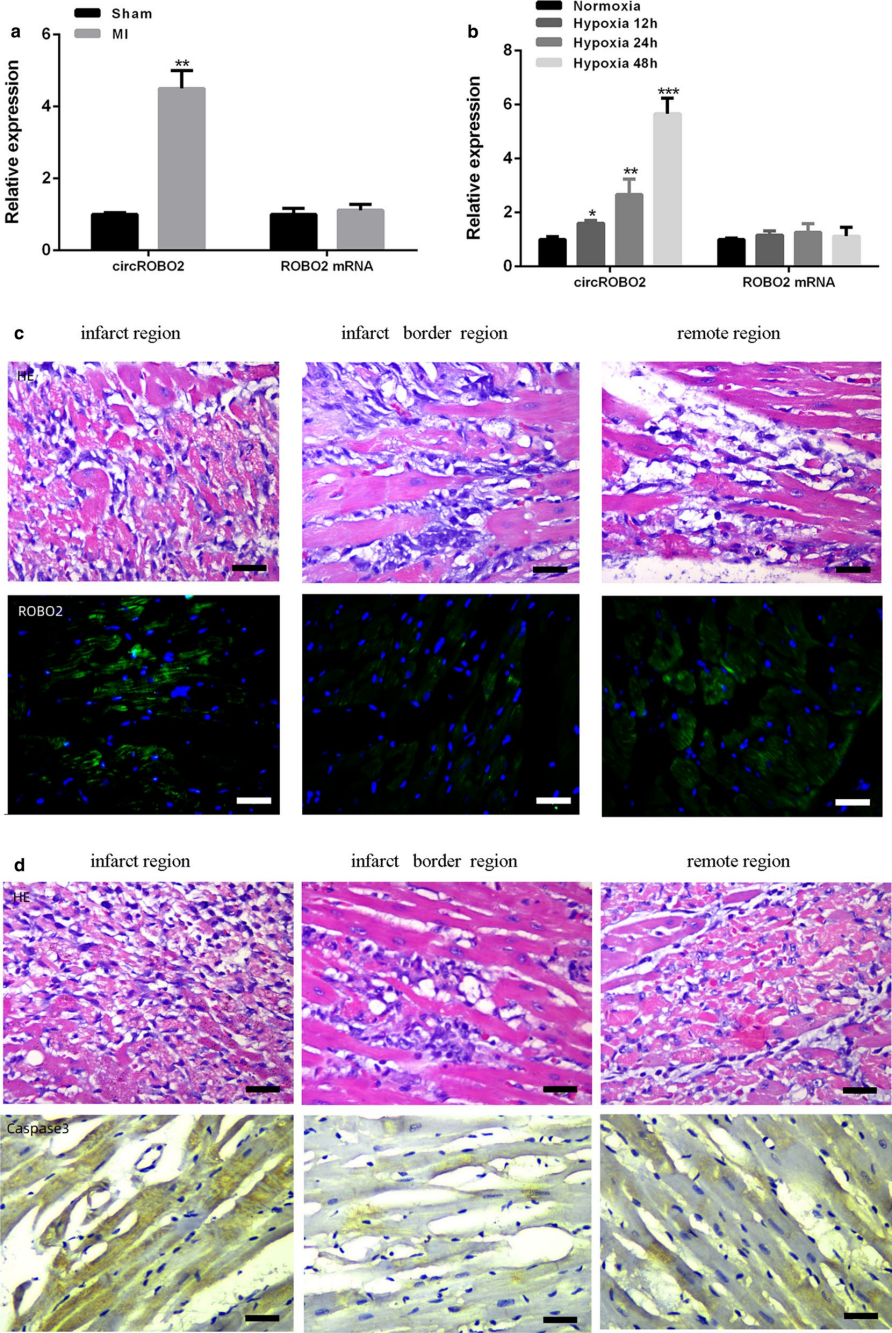
Ischemic injury induced the expression of circROBO2. **a** qRT-PCR to detect the relative expression levels of circROBO2 or ROBO2 mRNA in myocardium of MI and sham operation groups. **b** qRT-PCR to detect hypoxic treatment expression levels of circROBO2 or ROBO2 mRNA in primary cardiomyocytes. Experiment was repeated three times. *P < 0.05, **P < 0.01, ***P < 0.001. **c** HE&RNA ISH of circROBO2 in the infarct region, infarct border and the remote region from infarct, respectively. Scale bar: 30 µm. The green fluorescence signal indicated circROBO2. **d** HE&IHC of Capaspe3 in the infarct region, infarct border and the remote region from infarct, respectively. Scale bar: 30 µm

The cardiomyocyte morphology and infarct size were observed and measured. The expression levels of circROBO2 in MI models and hypoxic-treated primary myocardial cells were evaluated. As shown in Fig. 1a, compared with the sham operation group, circROBO2 was significantly up-regulated in myocardial tissues in MI mouse model (*P* < 0.05), while linear expression of ROBO2 was not significantly changed. Consistent with our results in ischemic myocardium, the expression levels of circROBO2 were gradually increased in a time-dependent manner in primary myocardial cells exposed to ischemic injury (*P* < 0.05). While the linear ROBO2 expression did not change (Fig. 1b). To detect the expression of circROBO2 post MI in situ, RNA ISH was used to detect the expression pattern of circROBO2 in myocardial tissues (Fig. 1c). The results indicated that circROBO2 was induced in the infarct region compared to the adjacent normal tissues, but was also slightly induced in the remote region (less than infarct region). In addition, Caspase3 staining indicated that cell apoptosis was also induced in the same region (Fig. 1d). These results indicated that circROBO2 played an important role in ischemic heart injury.

### CircROBO2 functioned as a sponge for miR-1184 to regulate TRADD in primary cardiomyocytes

**Fig. 2:**
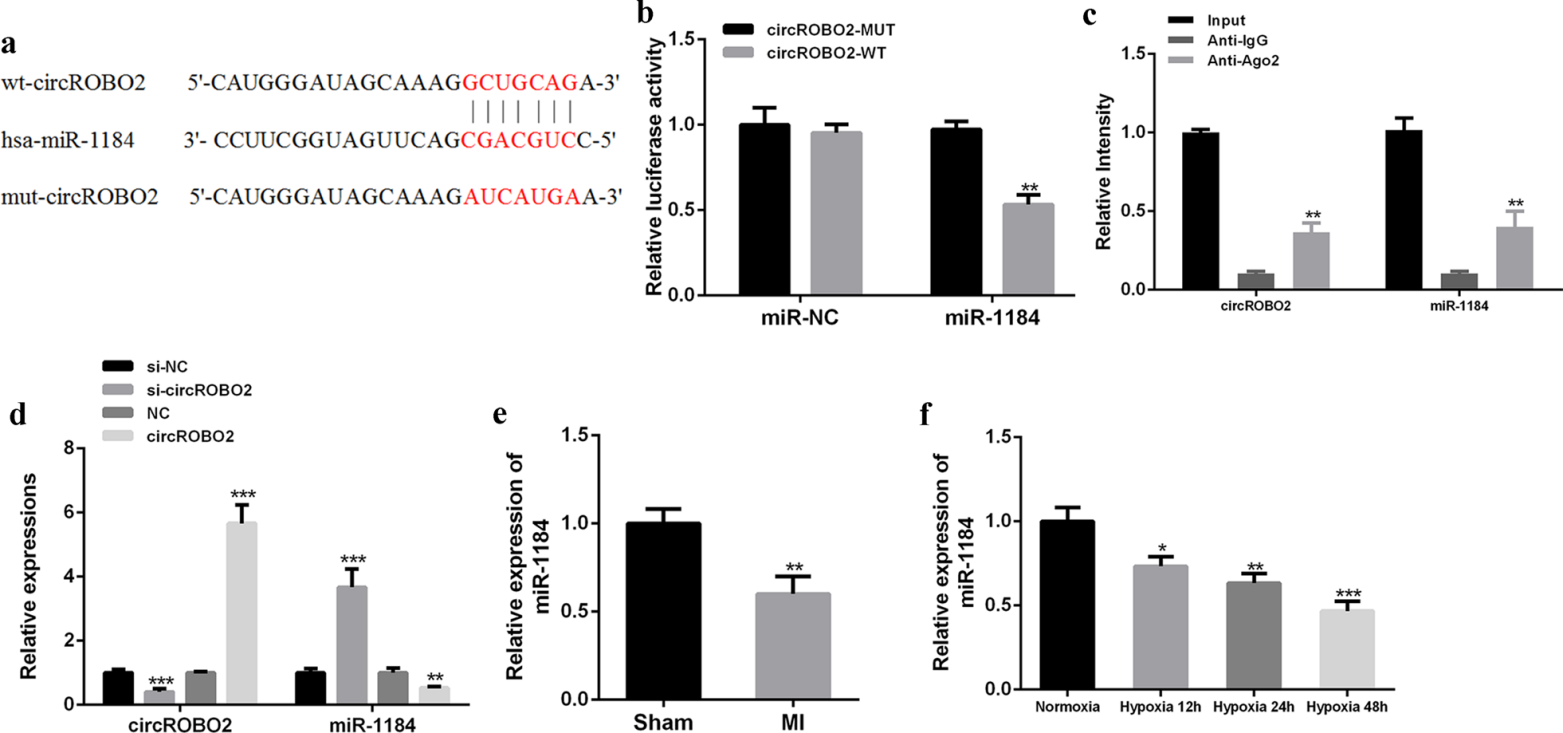
CircROBO2 played a biological role through miR-1184. **a** Circ Interactome predicted the putative targeting sites for circROBO2 and miR-1184. **b** Luciferase activity analysis. **c** The RIP method was used in primary cardiomyocytes. Determine the degree of enrichment of circROBO2 and miR-1184 RNA in the IP complex. Anti-immunoglobulin G (IgG) was used as a control. **d** Knockdown or overexpression of circROBO2 or miR-1184 in primary cardiomyocytes. **e** The expression levels of miR-1184 in ischemic myocardium and normal tissues. **f** The expression levels of miR-1184 in myocardial cells of mice exposed to normoxic or hypoxic conditions. All above the experiments were repeated three times. *P < 0.05, **P < 0.01, ***P < 0.001

Circ Interactome predicted that miR-1184 was a potential target of circROBO2 (Fig. 2a). To verify this, WT-circROBO2 or mutant-circROBO2 luciferase reporter plasmid was constructed for luciferin enzyme reporter gene assay. The results showed that the luciferase activity of pGL3-REPOR-circROBO2-WT was significantly reduced by co-transfecting miR-1184 mimics (*P* < 0.05), but the luciferase activity of pGL3-REPOR-circROBO2-mut did not change (Fig. 2b). WT-miR-1184 or MUT-miR-11849 (in the biding site with circROBO2) was also transfected into the pGL3-REPOR-circROBO2 reporter system. luciferase activity of WT-miR-1184 transfected system was significantly reduced, but not that of MUT-miR-1184. In addition, as shown in Fig. 2c, RIP analysis using anti-Ago2 in cell extracts found that circROBO2 and miR-1184 were preferentially enriched in miRNPs containing Ago2 compared with anti-IgG immunoprecipitation. As shown in Fig. 2e, compared with the sham group, the expression of miR-1184 was significantly down-regulated in myocardial tissues of the mice MI model (*P* < 0.05). As shown in Fig. 2d, compared with the control group, the expression levels of circROBO2 in the si-circROBO2 group were significantly reduced (*P* < 0.05), while the expression levels of circROBO2 were significantly increased in the circROBO2 group (*P* < 0.05). In the si-circROBO2 group, the expression levels of miR-1184 were significantly increased (*P* < 0.05), and the expression levels of miR-1184 were significantly decreased in the circROBO2 group (*P* < 0.05). Consistent with our results in ischemic myocardium, miR-1184 was detected in primary myocardial cells exposed to ischemic injury. The expression levels of miR-1184 were gradually decreased in primary cardiomyocytes induced by hypoxia (*P* < 0.05) (Fig. 2f). These results indicated that circROBO2 may exert its biological functions through miR-1184.

### CircROBO2 sponged miR-1184 to upregulate the expression of TRADD indirectly

**Fig. 3:**
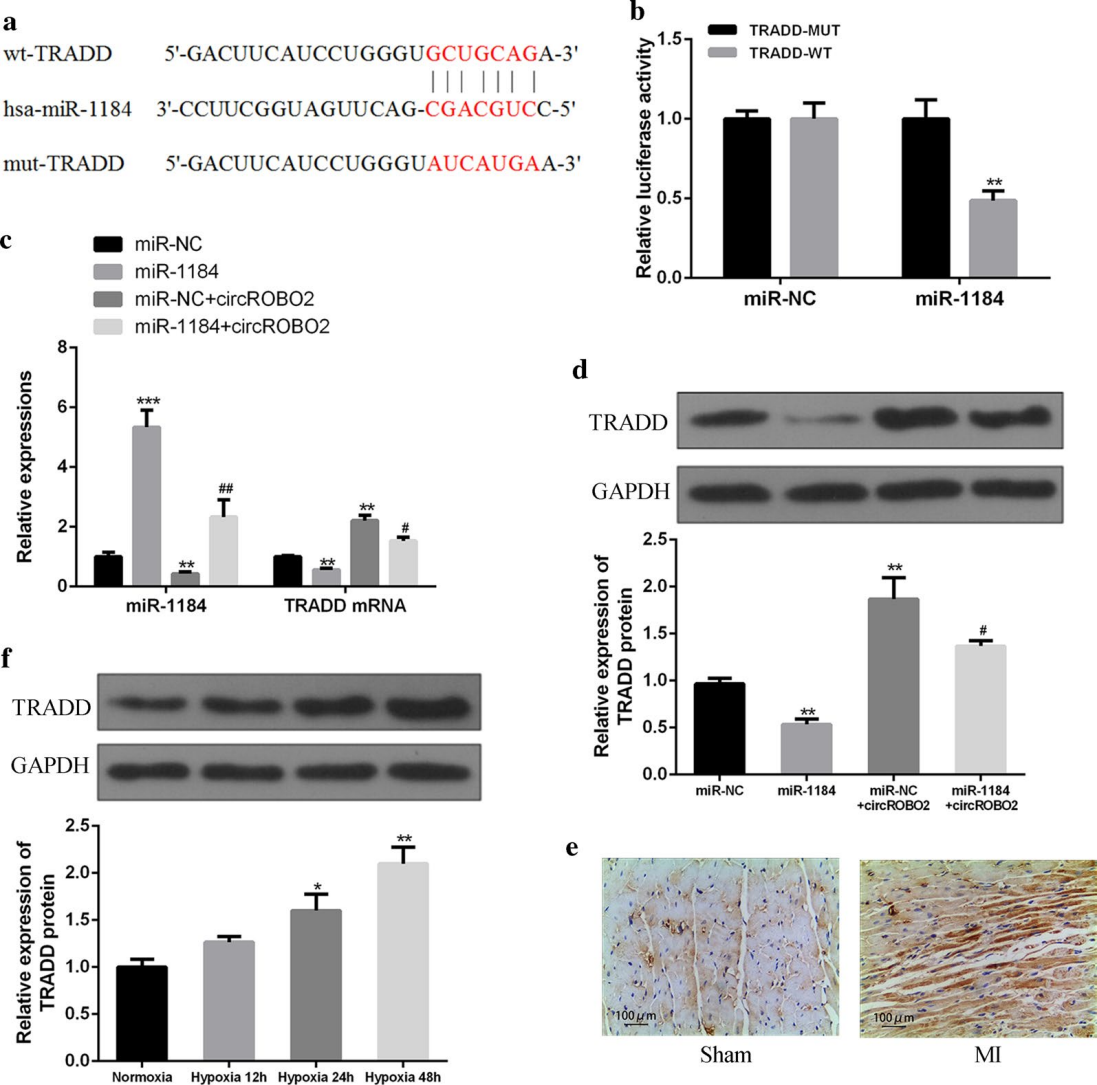
CircROBO2 up-regulated the expression of TRADD by sponging miR-1184. **a** Putative binding sites for miR-1184 and TRADD3′-UTR. **b** Analysis of luciferase activity of PGL-miR-1184 in HEK293 cells by co-transfecting withTRADD-3′-UTR-WT or TRADD-3′-UTR-Mut vector. The experiments were repeated three times. *P* < 0.05, **P < 0.01, ***P < 0.001. **c** TRADD mRNA expression levels were detected by co-transfecting miR-1184 mimics or miR-NC, circROBO2 vector or control vector in primary cardiomyocytes. The experiments were repeated three times. *P* < 0.05, **P < 0.01, ***P < 0.001. **d** Expression levels of TRADD protein in primary myocardial cells after co-transfecting with miR-1184 mimics, miR-NC + circROBO2 vector or miR-1184 + circROBO2. The experiments were repeated three times. **e** IHC analysis of caspase3 in myocardium form sham and MI group. **f** Expression levels of TRADD protein in cardiomyocytes of mice exposed to normoxic or hypoxic conditions (12, 24 and 48 h). There mice were used for this experiment. * vs control group, # vs circROBO2 group. **P < 0.01, ^##^*P* < 0.01

The online prediction tool Starbase v2.0 identified TRADD as a potential target of miR-1184 (Fig. 3a). To verify the prediction results, WT-miR-1184 or mutant (Mut) -miR-1184 fluorescein was used for enzyme reporter plasmid test. The results showed that the luciferase activity of pGL3-REPOR-TRADD-WT was reduced by miR-1184 mimic, but the luciferase activity of pGL3-REPOR-TRADD-Mut was not changed (Fig. 3b). In addition, as shown in Fig. 3c and d, compared with the control group, the expression of miR-1184 in the miR-1184 group was significantly up-regulated (*P* < 0.05), and the expression of miR-1184 in the circROBO2 group was significantly down-regulated (*P* < 0.05). Compared with the circROBO2 group, the expression of miR-1184 was significantly up-regulated after co-transfection of miR-1184 with circROBO2 (*P* < 0.05). Compared with the control group, the expression of TRADD was significantly down-regulated at both mRNA and protein levels in the miR-1184 group (*P* < 0.05), and the expression of TRADD in the circROBO2 group was significantly up-regulated at both mRNA and protein levels (*P* < 0.05). Compared with the circROBO2 group, the expression of TRADD was significantly down-regulated after co-transfection of miR-1184 with circROBO2 (*P* < 0.05). As shown in Fig. 3e, compared with the sham group, the expression of TRADD was significantly up-regulated in myocardium tissues of the mice MI model (*P* < 0.05). While in primary myocardial cells exposed to ischemic injury, the expression levels of TRADD were gradually increased (*P* < 0.05) (Fig. 3f). In summary, these results showed that circROBO2 up-regulated the expression of TRADD by acting as a sponge for miR-1184 in MI pathological process.

### Knockdown of circROBO2 inhibited myocardial apoptosis via the miR-1184/TRADD axis in vitro

**Fig. 4:**
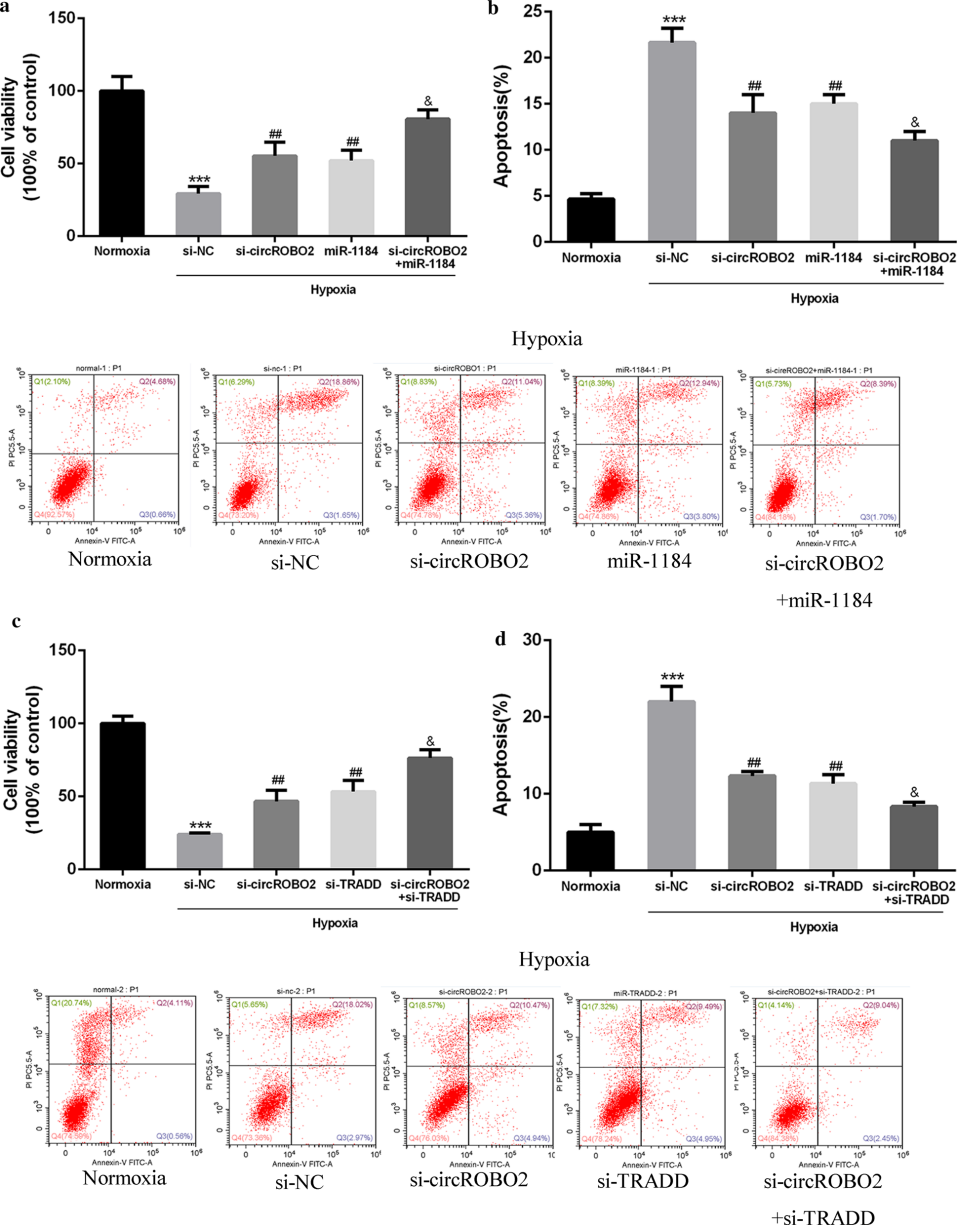
Biological effects of circROBO2 in hypoxic cardiomyocytes in vitro. **a**, **c** Cell viability analysis of mouse cardiomyocytes. Cardiomyocytes were exposed to normoxic or hypoxia combined with transfecting with si-NC, si-circROBO2 + miR-1184 mimics, si-circROBO2 + miR-1184 or si-circROBO2 + si-TRADD. **b**, **d** Cell dysfunction of mouse cardiomyocytes exposed to normoxic or hypoxia combined with transfecting with si-NC, si-circROBO2 + miR-1184 mimics, si-circROBO2 + miR-1184 or si-circROBO2 + si-TRADD. All above the experiments were repeated three times. * vs normoxia control group, # vs si-NC group, & vs si-circROBO2 group. ***P < 0.001, ^##^*P* < 0.01 and ^&^*P* < 0.05

The role of circROBO2 in myocardial cell viability and apoptosis was then explored. As shown in Fig. 4a, b, compared with the Normoxia group, hypoxia significantly inhibited cell viability in mouse cardiomyocytes. Compared with the si-NC group, si-circROBO2 and pcDNA-miR-1184 effectively enhanced the viability of mouse cardiomyocytes and effectively inhibited cell apoptosis. While co-transfection of si-circROBO2 with miR-1184 further enhanced the viability of cardiomyocytes and inhibited apoptosis. As shown in Fig. 4c, d, si-TRADD effectively enhanced the viability of mouse cardiomyocytes and effectively inhibited the apoptosis of cardiomyocytes. Co-transfection of si-circROBO2 with si-TRADD effectively reversed the effect of si-circROBO2 on cell viability. These results indicated that knockdown of circROBO2 enhanced cell viability and inhibited apoptosis of mouse cardiomyocytes by regulating the miR-1184/TRADD axis.

### Knockdown of circROBO2 improved myocardial function through up-regulating miR-1184 in vivo

**Fig. 5:**
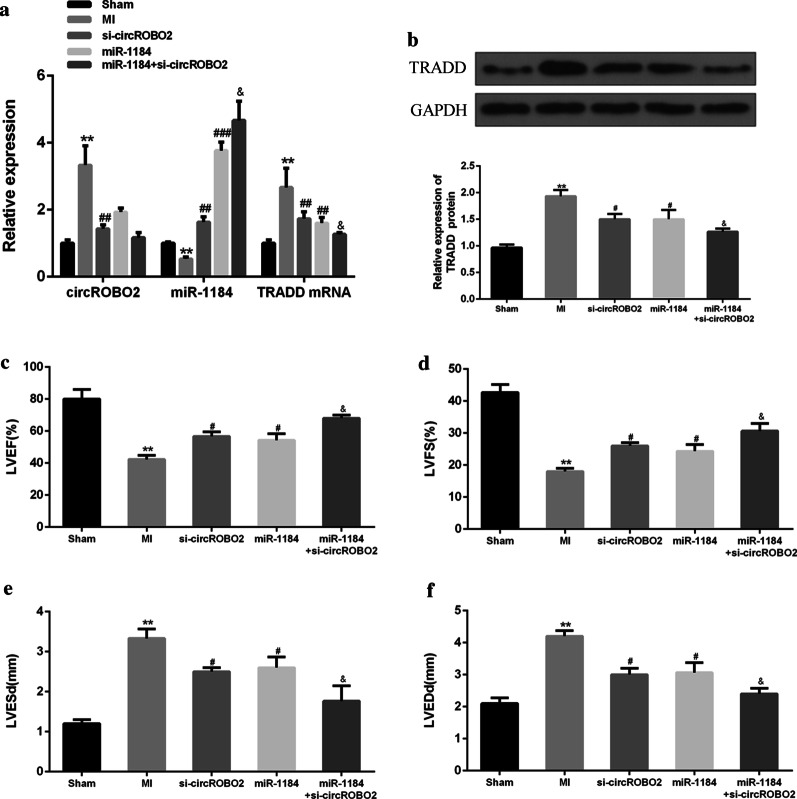
Effects of overexpression of circROBO2 on myocardial function. **a** Relative expression levels of circROBO2, miR-1184 and TRADD mRNA in mice group of sham, MI, si-circROBO2 transfection, miR-1184 transfection and miR-1184 + circROBO2 transfection. **b** TRADD protein expression levels. LVEF (**c**), LVFS (**d**), Expression of LVESd (**e**) and LVEDd (**f**) in Sham, MI, si-circROBO2, miR-1184, and si-circROBO2 + miR-1184 groups. Myocardial cells were from three individual mice. * vs normoxic control group, # vs NC group and vs circROBO2 group. ***P < 0.001, ^##^*P* < 0.01 and ^&^*P* < 0.05

Next, the role of knockdown of circROBO2 in MI mouse model was explored. As shown in Fig. 5a, b, transfection of si-circROBO2 and miR-1184 attenuated the expression of circROBO2 and TRADD in MI mouse model. The expression of miR-1184 was significantly up-regulated, while the expression levels of circROBO2 and TRADD were further reduced after co-transfection of si-circROBO2 with miR-1184. The expression levels of miR-1184 were further increased. Compared with the surgery group, LVEF and LVFS of the myocardial tissues in the MI treatment group were significantly reduced, while LVESd and LVEDd were significantly increased. In addition, overexpression of si-circROBO2 and miR-1184 significantly increased LVEF and LVFS. Furthermore, overexpression of si-circROBO2 and miR-1184 further reduced LCESd and LVEDd (Fig. 5c–f). These results indicated that overexpression of circROBO2 partially contributed to dysfunction after AMI by inhibiting miR-1184.

### Knockdown of circROBO2 alleviated myocardial injury and apoptosis by sponging miR-1184 in vivo

**Fig. 6:**
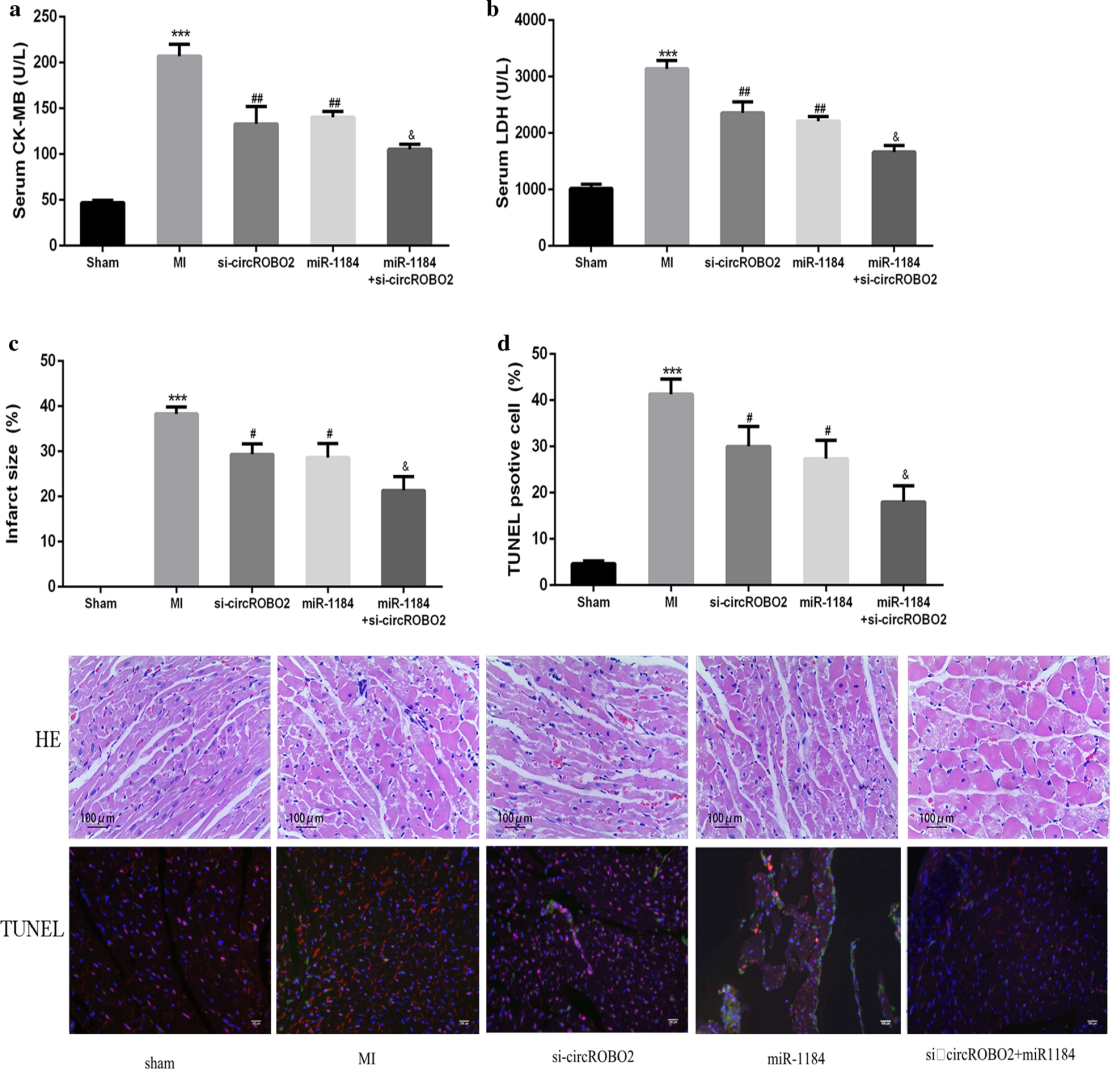
The effects of knockdown of circROBO2 on myocardial injury. Sham, MI, si-circROBO2, miR-1184 and si-circROBO2 + miR -1184 mice serum CK-MB (**a**) and LDH (**b**) and myocardial infarction size (**c**) were detected and compared. All above the experiments were repeated 3 times. * vs normoxia control group, # vs si-NC group, &vs si-circROBO2 group. ***P < 0.001, ^##^*P* < 0.01 and ^&^*P* < 0.05. **d** HE staining and TUNEL/nkx2.5 co-staining to detect the apoptosis of mouse cardiomyocytes. The red signaling indicate TUNEL, green signaling indicate nkx2.5, Scale bar: 100 μm

As shown in Fig. 6a, b, compared with the Sham group, the release of serum CK-MB and LDH was significantly increased in the MI group. The si-circROBO2 and miR-1184 group were able to significantly reduce the release of CK-MB and LDH. Co-transfection of miR-1184 with si-circROBO2 further enhanced the release of CK-MB and LDH. In addition, except for the Sham group, the other groups had different degrees of myocardial infarction. The area of myocardial infarction in the si-circROBO2 and miR-1184 overexpression group was significantly reduced, and co-transfection of miR-1184 with si-circROBO2 could further enhance the protective effect of the heart (Fig. 6c). As shown in Fig. 6d, TUNEL and Nkx2.5 (a marker of cardiomyocytes) co-staining showed that the apoptosis rate of cardiomyocytes in the MI group was increased significantly compared with the sham group in 1 week after (rAAV9)-mediated gene delivering in the heart, while the apoptosis rate of cardiomyocytes in the si-circMACF1 and miR-1184 overexpression groups was significantly reduced, and co-transfection of miR-1184 with si-circROBO2 could further reduce apoptosis in myocardium. HE staining results showed that the infarcted heart tissue had structural abnormalities of myofibrils, muscle fiber swelling, and necrotic lesions. In MI mice with the overexpression of si-circROBO2 or miR-1184, these pathological changes were significantly reduced. Co-transfection of miR-1184 with si-circROBO2 further weakened these pathological changes. These results indicated that circROBO2 effectively increased myocardial apoptosis through the miR-1184/TRADD axis.

## Discussion

AMI is one of the most critical and severe diseases in clinical practice, and is the main cause of high morbidity, high hospitalization rate and high mortality worldwide (Bourron et al. 2017; Li et al. 2017). The main pathogenesis of AMI is that the endothelial function is damaged, the atherosclerotic plaque is unstable, and a series of pathophysiological changes such as rupture and erosion occur, leading to platelet aggregation and activation. Internal and external systemic coagulation activates the excessive conversion of fibrinogen into fibrin, combines with a series of components such as white blood cells and red blood cells to form thrombus, therefore causing a large number of thrombus formation in the coronary artery. This further causes complete or incomplete blockage of the diseased coronary artery, resulting in reduced blood flow perfusion of myocardial tissues and thus myocardial infarction. Cardiovascular disease is one of the global human health problems (Shahrivari et al. 2017). To improve the diagnosis, treatment and curative effect of MI patients, and to improve the prognosis of cardiovascular patients, searching for early diagnosis biomarkers for AMI is still of great importance.

The mechanisms of AMI have been extensively explored. CircRNAs are stable and have strong specificity, and exert their functions by regulating gene expression, sponging miRNAs, regulating transcription and translation (Cui et al. 2016; Lasda and Parker 2016). Studies have shown that circRNAs play key roles in atherosclerosis, MI, myocardial fibrosis, atrial fibrillation and other cardiovascular diseases (Li et al. 2019b). An increasing number of circRNAs have been reported to regulate the apoptosis of myocardial cells (Zhou and Yu 2017). Studies have found up-regulated expression of cicRNA-Cdr1as in MI mouse models, in which Cdr1as up-regulates its target genes SP1 and PARP by binding to miR-7a, promoting cell apoptosis and triggering MI (Geng et al. 2016). The present study found that circROBO2 was up-regulated in myocardial tissues and primary myocardium cells exposed to ischemic injury. And si-circROBO2 enhanced cell viability and inhibited apoptosis. In vivo experiments showed that si-circROBO2 increased LVEF and LVFS, inhibited LVESd and LVEDd, reduced the levels of clear CK-MB and LDH, and reduced the area of myocardial infarction. Therefore, the development of AMI could be modulated by inhibiting the expression of circROBO2.

As the target of circRNAs, miRNAs can degrade or inhibit translation of target mRNAs. It has been reported that miRNAs play a part in cardiovascular system diseases (Zeng et al. 2018; He et al. 2018). MiRNAs have shown to be potential biomarkers for AMI and ischemia–reperfusion injury (I/R) (Gidlöf et al. 2013). For example, it was reported that miR-17-5p is up-regulated in brain injury and oxidative stress induced by acute infarction, and this up-regulation of miR-17-5p also induced apoptosis (Yang et al. 2018). Another study reported that the expression of miR-1184 is significantly up-regulated in colorectal cancer (Chen et al. 2016). Our study found that miR-1184 was the target gene of circROBO2. CircROBO2 was significantly up-regulated in myocardial tissues and primary cardiomyocytes exposed to ischemic damage. MiR-1184 effectively enhanced the viability of mouse cardiomyocytes and effectively inhibited the apoptosis of cardiomyocytes, while Co-transfection of si-circROBO2 with miR-1184 further enhanced the viability of cardiomyocytes and inhibited apoptosis. These findings indicated that circROBO2 may increase apoptosis of myocardial cells by regulating miR-1184.

Emerging evidence has shown that miRNAs interact with circRNAs to regulate certain pathways and affect disease development (Zhang and Yang 2018). TRADD is an intracellular aptamer molecule containing a death domain, mainly interacting with the membrane surface death receptor TNFRA combining pathways (Koo et al. 2950). TRADD can mediate apoptosis, and participate in intercellular signaling pathways (Pobezinskaya and Liu 2012). This study found that TRADD was a potential target for miR-1184. The expression levels of TRADD were significantly increased in myocardial tissues and primary cardiomyocytes exposed to ischemic injury. Si-TRADD effectively enhanced the viability of mouse cardiomyocytes and effectively inhibited apoptosis of cells. Co-transfection of si-circROBO2 with si-TRADD further enhanced the viability of cardiomyocytes and inhibited apoptosis. In addition, overexpression of miR-1184 inhibited the expression of TRADD, but overexpression of circROBO2 reversed the effect of overexpression of miR-1184 on the expression of TRADD in primary myocardial cells. In addition, overexpression of miR-1184 or downregulation of mir-1184 can partially reverse the protective effect of overexpression of circROBO2 on cardiomyocyte viability and apoptosis. It was further confirmed that knockdown of circROBO2 effectively reduced cardiomyocyte apoptosis by regulating the mir-1184/TRADD axis in vivo.

## Conclusion

Knockdown of circROBO2 can reduce myocardial cell apoptosis by regulating the miR-1184/TRADD axis. This suggests that circROBO2 may be a potential pathogenic gene for AMI, which would provide evidence for the clinical prognosis of this disease.

## Data Availability

The analyzed data sets generated during the study are available from the corresponding author on reasonable request.
